# Assessment of Spatial Variability of Heavy Metals in Metropolitan Zone of Toluca Valley, Mexico, Using the Biomonitoring Technique in Mosses and TXRF Analysis

**DOI:** 10.1155/2013/426492

**Published:** 2013-06-17

**Authors:** Graciela Zarazúa-Ortega, Josefina Poblano-Bata, Samuel Tejeda-Vega, Pedro Ávila-Pérez, Carmen Zepeda-Gómez, Huemantzin Ortiz-Oliveros, Guadalupe Macedo-Miranda

**Affiliations:** ^1^Environmental Science Division, National Institute for Nuclear Research, Carretera México-Toluca S/N, La Marquesa, 52750 Ocoyoacac, MEX, Mexico; ^2^Autonomous University of Mexico State, Unidad San Cayetano, 50294 Tlachaloya, MEX, Mexico; ^3^Technology Institute of Toluca, Apartado Postal 890, 52149 Metepec, MEX, Mexico

## Abstract

This study is aimed at assessing atmospheric deposition of heavy metals using the epiphytic moss genera *Fabronia ciliaris* collected from six urban sites in the Metropolitan Zone of the Toluca Valley in Mexico. The concentrations of K, Ca, Ti, V, Cr, Mn, Fe, Ni, Cu, Zn, Rb, Sr, and Pb were determined by total reflection X-ray fluorescence technique. Results show that the average metal concentration decrease in the following order: Fe (8207 mg/Kg) > Ca (7315 mg/Kg) > K (3842 mg/Kg) > Ti (387 mg/Kg) > Mn, Zn (191 mg/Kg) > Sr (71 mg/Kg) > Pb (59 mg/Kg) > Cu, V (32 mg/Kg) > Cr (24 mg/Kg) > Rb (13 mg/Kg) > Ni (10 mg/Kg). Enrichment factors show a high enrichment for Cr, Cu, Zn, and Pb which provides an evidence of anthropogenic impact in the industrial and urban areas, mainly due to the intense vehicular traffic and the fossil fuel combustion. Monitoring techniques in mosses have proved to be a powerful tool for determining the deposition of heavy metals coming from diverse point sources of pollution.

## 1. Introduction

Atmospheric pollution has become one of the most important issues of air quality due to its impact on human health and the environment. Suspended particulates are introduced into the atmosphere from a variety of natural and anthropogenic sources, although the latter are predominant in the urban and industrial areas. Some of the well known anthropogenic processes contributing to the airborne particulate pollution include transportation, industrial activities, biomass burning and agricultural activities. In the case of anthropogenic pollution, the particulate matter can contain metal trace element like Cr, Cu, Zn, Ni, Pb, and so forth [1].

Biomonitoring methods have become an important part of air pollution measurements. They are a widely available and effective means to determine “the levels of air pollutants and their impact on biological receptors” [2, 3] and therefore complement instrumental measurement methods. 

Due to their peculiar morphological and physiological characteristics mosses (division *Bryophytes*) are useful indicators of a wide range of contaminants. Bryophytes are poikilohydric species with a diverse means for water and mineral uptake [4]. As most bryophytes are small and the leaves of many mosses and folious liverworts consist of only one cell layer, the surface-to-volume ratio is high.

Bryophytes are resistant against many substances which are highly toxic for other plants. As a consequence of their nutrient cycling and uptake mechanisms they tend to accumulate pollutants. Bryophytes show several advantages as biomonitors of atmospheric deposition over other species, such as the lack of a protective cuticle and thick cell walls, numerous cell wall constituents with negatively charged groups, mineral nutrition obtained mainly from wet and dry deposition, and widespread distribution of several species together with simplicity and cheapness of biomonitoring methods [5, 6].

Mosses have been extensively employed over the past 30 years to monitor and map atmospheric deposition of contaminants, especially trace metals, in different parts of the world, due to the high correlation between deposition rates and levels in moss tissues [7–11].

Recently, these organisms are being used to determine patterns of variation across the regions, identify the main polluted areas, and develop the understanding of long-range transboundary pollution [12] because metals are present in much higher concentrations in mosses compared with the levels in precipitation samples, where the detection limit is usually too low and contamination problems can occur [13–16].

The aim of the present study was to evaluate atmospheric deposition of heavy metals using as indicator the epiphytic moss genera *Fabronia ciliaris* from the Metropolitan Zone of the Toluca Valley in Mexico applying the Total Reflection X-Ray Fluorescence Spectrometry (TXRF) technique.

## 2. Experimental

### 2.1. Sampling Area

The Metropolitan Zone of the Toluca Valley (MZTV) is located in the central region of Mexico, in the Estado de Mexico, between 19°05′ and 19°25′ north latitude and 99°20′ y 99°50′ west longitude, and is situated in an area known as the Mexican Highlands (2660 masl). Using Köppen's climatic classification [17], the Valley of Toluca presents a C(w2)(w)bi(g) climate, which corresponds to a subhumid temperate climate, with a long summer, winter rain of less than 5%, isothermal, and with maximum temperatures before the summer solstice. The MZTV is one of the five largest urban areas (1208 km^2^) in Mexico and is an important center for industrial and agricultural activity, with a high population density [18].

**Figure 1:**
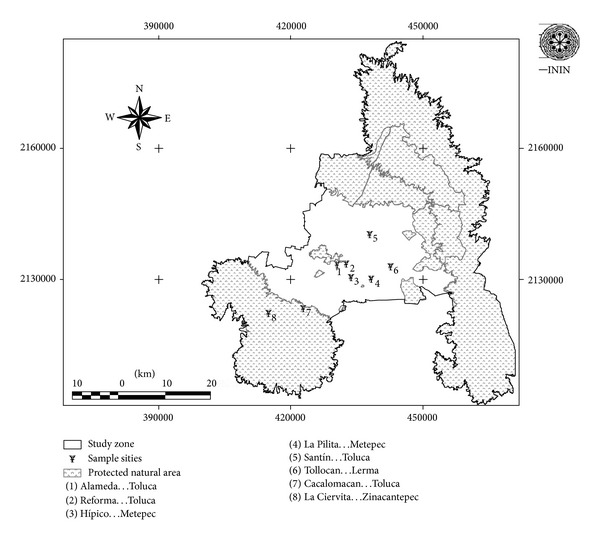
Geographical distribution of sampling locations at the Metropolitan Zone of the Valley of Toluca.

Six urban sites to the MZTV (Table 1; Figure 1) were selected taking into account the impact of industrial and urban pollution sources and the species of epiphytic moss *Fabronia ciliaris* being representative. At each sampling location 10 trees were selected and were taken 10 cm^2^ of epiphytic mosses; they were obtained at a height greater than one meter. Sampling was done in November 2010, that corresponding to dry-cold season.

### 2.2. Sample Preparation

The sample mosses were carefully washed with distilled water, freeze-dried, ground, sifted to 200 mesh, and homogenized. 0.3 g subsample of each site was putted in a closed digestion vessel was added 4 mL of HNO_3_ and allowed to pre-digest for 12 hours [19], then each of the samples was added with 1 mL of HCl and digested in a microwave furnace (CEM-MarsX): increasing the temperature in 30 min to 170°C, 300 psi, and 15 min of maintenance time [20]. Then, 1.5 mL of H_2_O_2_ was added to the sample before the second digestion under the same conditions as aforementioned.

The digested sample was transferred into a volumetric flask, the reaction vessels were washed out with deionized water, and the flask volume made up to a final volume of 10 mL. 100 *μ*L of Ge standard solution (Merck) of 20 *μ*g/mL was added as internal standard to 1 mL subsample of the digested samples and mixed. After that, 10 *μ*L of the digested and standardized sample was deposited on a silicon quartz glass holder, and dried under infrared light [21]. Only supra pure grade quality chemicals were used. All samples were digested in duplicate.

### 2.3. Sample Analysis

The TX 2000 X-Ray Spectrometer manufactured by Ital Structures was used in order to obtain the elemental concentration. A primary fine focus beam provided by the X-Ray tube with a molybdenum anode is monochromatized and is directed to the sample at a glancing angle less than the critical angle. The tube was operated at 40 kV and 30 mA. The fluorescent X-rays derived from the sample were detected with a solid state lithium-drifted silicon detector of 20 mm^2^ front area, cooled with liquid nitrogen. The energy resolution (FWHM) of the Si(Li) detector was 140 eV for Mn K*α* and its beryllium window was 8 *μ*m thick.

Three replicates were analyzed for each sample in order to evaluate the reproducibility of measurements. All samples were excited for 500 s. Heavy metal concentration was determined by internal standardization; this method is appropriate for a multielement determination and is normally used for TXRF. It is based on the sensitivity values of the different elements. For the analysis of the spectra and metal quantification, the software EDXRF32 was used [22]. Method detection limit (MDL) listed in Table 3 was calculated with the following formula [23]:

(1) $$\begin{array}{c} \text{MDL}=3\left(\frac{C_{x}}{I_{x}}\right)\left(\sqrt{2}I_{\text{BG}}\right), \end{array}$$

where MDL is method detection limit (minimum detectable mass mg/Kg); *C*_*x*_ is analyte concentration (mg/Kg); *I*_*x*_ is analyte net intensity (counts); and *I*_BG_ is background intensity (counts).

### 2.4. Enrichment Factors

Enrichment factors (EFs) were also investigated. This factor estimates the enrichment of K, Ca, V, Cr, Mn, Fe, Ni, Cu, Zn, and Pb by terrestrial or anthropogenic effects using (2). Titanium was selected as conservative element [24]. Values of reference soil, coming from two sites not impacted by anthropogenic activities [25], Cacalomacan and Ciervita (Figure 1), were considered as reference values. Consider

(2) $$\begin{array}{c} EF=\frac{{\left(M_{s}/{Ti}_{s}\right)}_{\text{moss value}}}{{\left(M_{o}/{Ti}_{o}\right)}_{\text{reference soils value}}}, \end{array}$$

where **EF** is enrichment factor; **M**_*s*_ is concentration of metal “*x*” in the moss sample; **T** **i**_*s*_ is concentration of conservative reference element (Ti) in the moss sample; **M**_*o*_ is concentration of metal “*x*” in reference soils; and **T** **i**_*o*_ is concentration of conservative reference element (Ti) in reference soils.

In order to know the enrichment level by metals, several criteria were considered, as it appears in Table 2. Ratio values above 10.0 are generally considered to reflect enrichment of the element in mosses [26–28].

### 2.5. Statistical Methods

Data were processed by the *Statgraphics Plus 5* program in order to obtain the average values, standard deviations, and confidence limits. The Statgraphics software allowed investigation of the existence of a normal distribution for the data values. The analysis of variance (ANOVA) was applied to data values when normal distribution was observed, and the Kruskal-Wallis test was used when no normal distribution law was observed, in order to deduce spatial distributions for heavy metals in the MZTV. The statistical methods were performed with a 95% confidence interval (CI; *α* = 0.05).

## 3. Results and Discussion

The absence of a significant contamination during the digestion process of the samples was checked using blanks (2 for each digestion series of 14 samples) containing only supra pure grade quality acids. In order to evaluate the accuracy and reproducibility of the analytical results, three subsamples of the reference material “IAEA-336 Lichen” from the International Atomic Energy Agency (IAEA) were analyzed. The elemental concentrations in the samples are shown in Table 3. As it can be seen, the measured concentrations were consistent with the certified values. Accuracy being measured as the percentage of recoveries (% R) after the acid digestion (ratio between values measured and certified in the reference material) was higher than 90% and the percentage relative error was from lower than 1% to 9%. The relative standard deviation for all elements was lower than 10%. Method detection limits (MDLs) for the experimental conditions are also shown.

**Figure 2:**
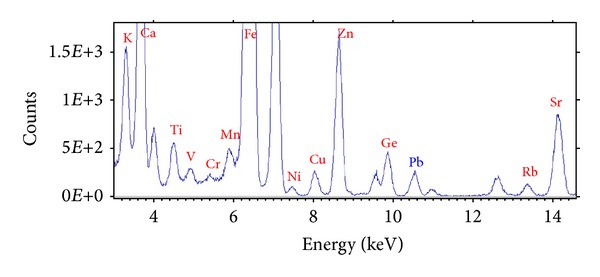
TXRF spectrum of a moss sample from Tollocan sampling site (6) at the Metropolitan Zone of the Valley of Toluca.

The K-lines of K, Ca, Ti, V, Cr, Mn, Fe, Ni, Cu, Zn, Rb, Sr, and L-lines of Pb were identified in moss samples.  Figure 2 shows the TXRF spectrum of a moss sample in site 6 (Tollocan).

Mean and standard deviation of the analyzed metals in the mosses are given in Table 4. The elements that showed the highest mean concentrations were Fe > Ca > K > Ti > Zn > Mn > Pb > Sr > Cu > V > Cr > Rb > Ni. In general the elements determinate present a variation in the concentration between different sampling sites (statistical significant differences, *P* < 0.05).

K, Ca, Ti, Mn, Fe, Rb, and Sr are metals mainly associated with terrigenous sources, so their presence in airborne particulate matter in the study area may be due primarily to resuspension of soil, road dust, agricultural livestock, forest fires, and so forth [24, 28, 29]. On the other hand V, Cr, Ni, Cu, Zn, and Pb in the MZTV can be associated mainly with anthropogenic sources, because even when these elements may be present naturally in soils they are considered like trace elements [30]; their presence in airborne particulate matter of the MZTV may be due to mobile sources such as combustion products of oils, lubricants and additives for automotive, tire wear, brake, and stationary sources such as automotive, metalworking, smelter, and chromium industries established in the MZTV [28, 31].

In general, concentrations of Cr, Cu, Zn, and Pb (Table 4) are the highest in sites Alameda, Reforma, and Tollocan (statistical significant differences, *P* < 0.05), and additionally Tollocan presented a highest metal concentration sum compared to the other sampling sites (statistical significant differences, *P* < 0.05), which can be related to traffic. It is known that the traffic is the main local source of contamination by metals like V, Cr, Cu, Zn, and Pb, who can be associated with traffic emissions and other vehicular sources, such as combustion of fossil fuels, the wear of tires, brake linings, and asphalt [6, 28, 32]. Similar behavior in the concentration of these elements could be associated with all sites settled on primary roads with high traffic, an average of 53,000 vehicles/day [18].

Alameda and Reform showed a similar behavior; they are urban parks located in the center of the Toluca City, and these sampling sites presented the highest lead concentration which can be associated with high buildings surrounding these parks who can contribute to this trend by obstructing pollution dispersion (so called resuspension phenomenon) of lead, copper, chromium, and zinc particles from metal-mechanic and smelter industries located in the industrial zone of Toluca [18].

Tollocan and Santin presented the highest sum element concentration; both sampling sites are located near the industrial area of Lerma City and roads with high traffic of vehicles using gasoline and diesel, an average 85000 vehicles/day [18]. Ti, Fe, and Mn may also have terrigenous source, as they are more exposed to the deposition of particles from soil resuspension and transport of fugitive dust because there are no prominent topographic barriers.

The enrichment factors determined in this study in relation to the composition of reference soils (Table 5) showed that elements Ca, V, Mn, Fe, and Ni are conservatives (terrigenous origin) typical constituents of soils included in content of aerosols [33], may originate from resuspension of soil and road dust, but also may be derived partly from some anthropogenic sources.

According to the criteria proposed K and Cr show a slightly-moderately enriched (Table 6) indicated that the sources were mainly pedological-soil or substrate contribution and anthropogenic activities, while high EF for Cu, Zn, and Pb pointed to a predominantly anthropogenic origin (EF > 10).

Alameda and Reform presented the highest lead EF; both are urban parks located in the center of the city of Toluca with high vehicular traffic, while in Tollocan site, the mosses presented high Cu, Zn, and Pb enrichment, these elements are considered as indicators of emission from fossil fuel combustion processes, including vehicle exhausts; so these sites may pose a risk to people living or working in these areas, because the exposure to long- and short-term particulate matter (≤PM_10_) can cause decreased lung function, which contributes to the presence of chronic respiratory diseases and premature death; on the other hand, according to the results of recent studies by the Institute of Health of the State of Mexico in the MZTV, the climate, altitude, wind circulation, and Urban and industrial growth, among others, make this area emphasize the likelihood of having high concentrations of small suspended particles.

## 4. Conclusions

The accuracy related to the sample preparation and the measurement with the TXRF equipment was acceptable (R: 91%–105%), which was demonstrated by the use of the standard reference material. In relation to the reproducibility the results show a good standard deviation (SD < 10%).

TXRF was used to measure a wide range of elements in the epiphytic moss, which has the ability to accumulate metals and therefore can be used as a bioindicator of anthropogenic activities in polluted metropolitan zones, since this organism is sensible to the variations of elemental concentrations in the atmosphere.

The analysis of moss does provide information about the presence of contaminants in the atmosphere, their spatial patterns of distribution, and how they are taken up by live organisms.

Most of the data concentrations in the *Fabronia ciliaris* of the sampling sites Alameda (1), Reforma (2), and Tollocan (3) were higher than concentrations in the other sampling sites due to the level of pollution in the atmosphere.

## Figures and Tables

**Table 1:** Description of sampling locations at the Metropolitan Zone of the Toluca Valley.

Reference	Sampling site	Geographic location		Altitude masl	Description
		North latitude	West longitude		
Alameda	1	19°17′23′′	99°39′43′′	2662	Urban park with high traffic
Reforma	2	19°17′27′′	99°38′29′′	2644	Urban park with high traffic
Hipico	3	19°15′50′′	99°37′54′′	2668	Urban park with high traffic
Pila	4	19°15′36′′	99°35′15′′	2623	Urban park with medium traffic
Santin	5	19°21′9′′	99°35′31′′	2596	Urban area with medium traffic
Tollocan	6	19°17′10′′	99°32′49′′	2589	Urban, industrial, and highway with the highest traffic
Cacalomacan	7	19°11′57′′	99°44′04′′	3100	Ecological park without urban settlements, nondisturbed by pollution
Ciervita	8	19°11′17′′	99°48′35′′	3317	Natural protected area without urban settlements, non-disturbed by pollution

**Table 2:** Criteria for evaluated moss enrichment factor.

Enrichment factor	Enrichment level
≤2	Conservative
3–5	Slightly enriched
6–9	Moderately enriched
≥10	Highly enriched

**Table 3:** Comparison between measured and reference elements concentration for the IAEA: Standard reference material: IAEA-336 Lichen.

Element	Reference values		Measured values		R	MDL
	Mean Concentration	SD	Mean Concentration	SD	%	mg/Kg
	mg/Kg (*n* = 9)			
K	1840	±200	1745	±31	95	12
Ca	NR	NR	2253	±172	NC	12
Ti	NR	NR	14.77	±0.87	NC	3.5
V	1.47	±0.22	1.37	±0.12	93	4.0
Cr	1.06	±0.17	0.96	±0.10	91	1.8
Mn	63	±7	62.54	±2.20	99	1.5
Fe	430	±50	396	±22	92	1.2
Ni	NR	NR	ND	NC	NC	NC
Cu	3.6	NR	3.64	±0.21	101	1.3
Zn	30.4	±3.4	34.88	±0.76	105	1.3
Rb	1.76	±0.22	1.70	±0.06	97	0.23
Sr	9.3	±1.1	9.35	±0.16	101	0.23
Pb	4.9	±0.6	4.76	±0.04	97	0.50

SD: standard deviation; R: recovery; NR: not reported; MDL: method detection limited; ND: not detected; NC: not calculated.

**Table 4:** Mean concentrations (mg kg^−1^) of elements in *Fabronia ciliaris. *

Element		Site 1 Alameda	Site 2 Reforma	Site 3 Hípico	Site 4 Pila	Site 5 Santin	Site 6 Tollocan
		Concentration in mg/Kg (*n* = 6)					
K	Mean	2824	4212	3787	4147	4771	4570
	SD	138	115	308	234	240	254
Ca	Mean	4818	4589	5993	3880	10245	9275
	SD	367	196	551	182	471	450
Ti	Mean	372	338	288	346	516	458
	SD	29	10	17	16	18	22
V	Mean	29.20	34.22	19.23	27.23	23.02	90.92
	SD	2.07	2.75	1.57	2.40	1.80	3.12
Cr	Mean	33.34	27.21	22.32	19.93	21.47	28.50
	SD	2.10	0.80	1.15	1.12	1.46	1.20
Mn	Mean	172	119	132	161	292	214
	SD	9	2	9	8	10	12
Fe	Mean	9332	6746	5616	6432	9562	10708
	SD	696	191	310	214	232	344
Ni	Mean	10.88	11.08	7.40	6.20	8.65	19.04
	SD	1.16	0.95	0.80	0.70	0.63	1.28
Cu	Mean	32.12	40.20	26.17	20.68	27.36	66.78
	SD	2.26	0.86	1.00	0.81	1.38	2.58
Zn	Mean	145	179	151	136	220	406
	SD	12	4.24	3	4	16	20
Rb	Mean	13.40	10.40	11.74	10.78	20.70	14.01
	SD	0.50	0.70	0.73	0.64	1.00	1.00
Sr	Mean	40.54	41.01	67.00	45.80	49.14	109.00
	SD	2.90	1.36	6.00	5.11	3.87	7.67
Pb	Mean	112	107	32.60	25.31	20.36	70.30
	SD	5	2.38	2.26	1.60	1.51	3.81

Sum element concentration		17934	16543	16154	15254	25777	25999

SD: standard deviation.

**Table 5:** Mean concentration in reference soils at the Metropolitan Zone of the Toluca Valley.

Concentration in mg kg^−1^ (*n* = 36)	
Element	Mean
K	6633
Ca	19563
Ti	3767
V	95
Cr	51
Mn	852
Fe	30968
Ni	30
Cu	12
Zn	86
Rb	NR
Sr	NR
Pb	15

NR: not reported; Soil: values obtained from Tejeda et al., 2012 [34].

**Table 6:** Moss enrichment factors (EFs) from samples sites at the Metropolitan Zone of the Toluca Valley.

Element	Site 1 Alameda	Site 2 Reforma	Site 3 Hípico	Site 4 Pila	Site 5 Santin	Site 6 Tollocan	EF mean
K	5	7	7	7	6	6	**6**
Ca	2	2	3	2	4	4	**3**
V	3	4	2	3	2	6	**3**
Cr	7	5	5	4	3	5	**5**
Mn	2	1	2	2	2	2	**2**
Fe	3	2	2	2	2	3	**2**
Ni	4	4	1	2	2	5	**3**
Cu	26	33	24	18	16	45	**27**
Zn	17	21	20	17	19	39	**22**
Pb	76	72	24	18	10	39	**40**

## References

[1] Barandovski L, Cekova M, Frontasyeva MV (2008). Atmospheric deposition of trace element pollutants in Macedonia studied by the moss biomonitoring technique. *Environmental Monitoring and Assessment*.

[2] Mulgrew A, Williams P (2000). *Biomonitoring of Air Quality Using Plants*.

[3] Boquete MT, Fernández JA, Aboal JR, Carballeira A (2011). Analysis of temporal variability in the concentrations of some elements in the terrestrial moss *Pseudoscleropodium purum*. *Environmental and Experimental Botany*.

[4] Zechmeister HG, Grodziñska K, Szarek-Cukaszewska G, Markert BA, Breure AM, Zechmeister HG (2003). Bryophytes. *Bioindicators and Biomonitors*.

[5] Tyler G (1990). Bryophytes and heavy metals: a literature review. *Botanical Journal of the Linnean Society*.

[6] Tremper AH, Agneta M, Burton S, Higgs DEB (2004). Field and laboratory exposures of two moss species to low level metal pollution. *Journal of Atmospheric Chemistry*.

[7] Berg T, Royset O, Steinnes E (1995). Moss (*Hylocomium splendens*) used as biomonitor of atmospheric trace element deposition: estimation of uptake efficiencies. *Atmospheric Environment*.

[8] Berg T, Steinnes E (1997). Use of mosses (*Hylocomium splendens* and *Pleurozium schreberi*) as biomonitors of heavy metal deposition: from relative to absolute deposition values. *Environmental Pollution*.

[9] Rühling A (1994). Atmospheric heavy metal deposition in Europe. *Estimations Based on Moss Analysis*.

[10] Gerdol R, Bragazza L, Marchesini R (2000). Monitoring of heavy metal deposition in Northern Italy by moss analysis. *Environmental Pollution*.

[11] Grodzinska K, Szarek G, Godzik B (1990). Heavy metal deposition in polish national parks—changes during ten years. *Water, Air, and Soil Pollution*.

[12] Harmens H, Norris DA, Koerber GR, Buse A, Steinnes E, Rühling Å (2008). Temporal trends (1990–2000) in the concentration of cadmium, lead and mercury in mosses across Europe. *Environmental Pollution*.

[13] Markert B, Herpin U, J Berlekamp C (1996). A comparison of heavy metal deposition in selected Eastern European countries using the moss monitoring method, with special emphasis on the ‘Black Triangle’. *Science of the Total Environment*.

[14] Markert B, Reus U, Herpin U (1994). The application of TXRF in instrumental multielement analysis of plants, demonstrated with species of moss. *Science of the Total Environment*.

[15] Fernández JA, Rey A, Carballeira A (2000). An extended study of heavy metal deposition in Galicia (NW Spain) based on moss analysis. *Science of the Total Environment*.

[16] Aboal JR, Fernández JA, Boquete T, Carballeira A (2010). Is it possible to estimate atmospheric deposition of heavy metals by analysis of terrestrial mosses?. *Science of the Total Environment*.

[17] Miller A (1982). *Climatología*.

[18] Secretaria del Medio Ambiente (2007). *Aire limpio: Programa para el Valle de Toluca 2007–2011*.

[19] Carballeira A, López J (1997). Physiological and statistical methods to identify background levels of metals in aquatic bryophytes: dependence on lithology. *Journal of Environmental Quality*.

[20] CEM (2001). *Methods for Microwave Accelerated Reaction System for Acid Digestion*.

[21] Deutsches Institut für Normung EV Total reflection x-ray fluorescence analysis (TXRF).

[22] Ital-Structures

[23] Klockenkämper R (1997). *Total-Reflection X-Ray Fluorescence Analysis*.

[24] Salomons W, Förstner U (1984). *Metals in the Hidrocycle*.

[25] Bargagli R, Brown DH, Nelli L (1995). Metal biomonitoring with mosses: procedures for correcting for soil contamination. *Environmental Pollution*.

[26] Lantzy RJ, Mackenzie FT (1979). Atmospheric trace metals: global cycles and assessment of man’s impact. *Geochem Cosmochem Acta *.

[27] Dragović S, Mihailović N (2009). Analysis of mosses and topsoils for detecting sources of heavy metal pollution: multivariate and enrichment factor analysis. *Environmental Monitoring and Assessment*.

[28] Aničić M, Frontasyeva M, Tomašević M, Popović A (2007). Assessment of atmospheric deposition of heavy metals and other elements in Belgrade using the moss biomonitoring technique and neutron activation analysis. *Environmental Monitoring and Assessment*.

[29] Chapman PM, Wang F, Janssen CR, Goulet RR, Kamunde CN (2003). Conducting ecological risk assessments of inorganic metals and metalloids: current status. *Human and Ecological Risk Assessment*.

[30] Ure A, Berrow M (1982). *The Chemical Constituents of Soils*.

[31] Monaci F, Moni F, Lanciotti E, Grechi D, Bargagli R (2000). Biomonitoring of airborne metals in urban environments: new tracers of vehicle emission, in place of lead. *Environmental Pollution*.

[32] Oliva SR, Rautio P (2004). Could ornamental plants serve as passive biomonitors in urban areas?. *Journal of Atmospheric Chemistry*.

[33] Malm WC, Sisler JF, Huffman D, Eldred RA, Cahill TA (1994). Spatial and seasonal trends in particle concentration and optical extinction in the United States. *Journal of Geophysical Research*.

[34] Tejeda S, Zarazúa G, Astivia F (2012). Geochemical analysis of soils from metropolitan zone of the Toluca Valley.

